# Comparative Analysis of RNA Families Reveals Distinct Repertoires for Each Domain of Life

**DOI:** 10.1371/journal.pcbi.1002752

**Published:** 2012-11-01

**Authors:** Marc P. Hoeppner, Paul P. Gardner, Anthony M. Poole

**Affiliations:** 1 Science for Life Laboratory, Department of Medical Biochemistry and Microbiology, Uppsala University, Uppsala, Sweden; 2 Biomolecular Interaction Centre & School of Biological Sciences, University of Canterbury, Christchurch, New Zealand; University of Texas at Austin, United States of America

## Abstract

The RNA world hypothesis, that RNA genomes and catalysts preceded DNA genomes and genetically-encoded protein catalysts, has been central to models for the early evolution of life on Earth. A key part of such models is continuity between the earliest stages in the evolution of life and the RNA repertoires of extant lineages. Some assessments seem consistent with a diverse RNA world, yet direct continuity between modern RNAs and an RNA world has not been demonstrated for the majority of RNA families, and, anecdotally, many RNA functions appear restricted in their distribution. Despite much discussion of the possible antiquity of RNA families, no systematic analyses of RNA family distribution have been performed. To chart the broad evolutionary history of known RNA families, we performed comparative genomic analysis of over 3 million RNA annotations spanning 1446 families from the Rfam 10 database. We report that 99% of known RNA families are restricted to a single domain of life, revealing discrete repertoires for each domain. For the 1% of RNA families/clans present in more than one domain, over half show evidence of horizontal gene transfer (HGT), and the rest show a vertical trace, indicating the presence of a complex protein synthesis machinery in the Last Universal Common Ancestor (LUCA) and consistent with the evolutionary history of the most ancient protein-coding genes. However, with limited interdomain transfer and few RNA families exhibiting demonstrable antiquity as predicted under RNA world continuity, our results indicate that the majority of modern cellular RNA repertoires have primarily evolved in a domain-specific manner.

## Author Summary

In cells, DNA carries recipes for making proteins, and proteins perform chemical reactions, including replication of DNA. This interdependency raises questions for early evolution, since one molecule seemingly cannot exist without the other. A resolution to this problem is the RNA world, where RNA is postulated to have been both genetic material and primary catalyst. While artificially selected catalytic RNAs strengthen the chemical plausibility of an RNA world, a biological prediction is that some RNAs should date back to this period. In this study, we ask to what degree RNAs in extant organisms trace back to the common ancestor of cellular life. Using the Rfam RNA families database, we systematically screened genomes spanning the three domains of life (Archaea, Bacteria, Eukarya) for RNA genes, and examined how far back in evolution known RNA families can be traced. We find that 99% of RNA families are restricted to a single domain. Limited conservation within domains implies ongoing emergence of RNA functions during evolution. Of the remaining 1%, half show evidence of horizontal transfer (movement of genes between organisms), and half show an evolutionary history consistent with an RNA world. The oldest RNAs are primarily associated with protein synthesis and export.

## Introduction

Following demonstration that RNA can act as genetic material [1]–[3] and biological catalyst [4], [5], the study of the origin and early evolution of life on Earth has been heavily focused on the potential for an RNA world. The RNA world hypothesis is that RNA was both genetic material and main biological catalyst, prior to the advent of DNA and templated protein synthesis [6]–[8]. The chemical plausibility of an RNA world has been intensively investigated through the application of in vitro methodologies that enable selection and subsequent characterization of novel RNA functionalities [9], [10]. Equally, the discovery of naturally-occurring functional RNAs in biological systems has expanded our understanding of the ways in which extant organisms utilize this macromolecule in a wide range of contexts, including catalysis, regulation, and as sequence-based guides [11]–[15].

A central tenet of RNA world theory as an account of the early evolution of life on Earth is the Principle of Continuity [6], namely, that modern systems are the product of gradual evolution from earlier states. Consequently, it is possible that some RNA families could be direct descendants of molecules that first evolved in the RNA world [16], [17]. The broad functionality of RNA both in terms of catalysis and biological function hints at a possibly complex RNA world [12], [17], [18], but assessing the antiquity of individual RNA families has been hampered by limited comparative data, and difficulties in annotating RNAs in genomes [19]. At the same time, it seems likely that many RNA families significantly postdate the RNA world, having evolved de novo much later in the evolution of life [13], [20]. Indeed, for protein-coding genes, both very deep evolutionary histories [21]–[23] and more recent origins [24], [25] have been established.

Assigning relic status to individual RNAs is not without significant complication. First, placing RNAs with non-universal distributions into the common ancestor of archaea, bacteria and eukaryotes requires lineage or domain-specific losses to be invoked [26]. While loss is plausible, it is difficult to verify at the level of cellular domains, since recent origin versus lineage-specific loss following a more ancient origin cannot be readily distinguished, and other data must be considered [27], [28]. Another process that may obfuscate the history of early RNA-based life is the propensity for genes to undergo horizontal transmission, from a donor to a recipient. For protein-coding genes, there is now overwhelming evidence that horizontal gene transfer is a significant evolutionary force, particularly for microbes [29], [30]. Consequently, gene-based phylogenies do not always provide an accurate means of gauging the evolutionary history of species, and, extrapolating across the tree of life and several billion years of evolutionary history, it is plausible that no gene will have remained untouched by horizontal gene transfer [31]. Consequently, historical signal consistent with RNA world continuity may have been erased through subsequent gene transfer events. Conversely, effective spread by horizontal transmission could lead to RNAs appearing artificially ancient. Finally, many RNAs may be more recent evolutionary innovations, and may not be RNA world relics [13].

These concerns notwithstanding, it remains commonplace for novel RNAs or RNA families to be discussed in regard to their potential relevance to the RNA world. Indeed, there are countless qualitative surveys derived from review of the experimental literature (see for example [11], [12], [14], [17], [18], [32]), which often extrapolate deep evolutionary origins from limited comparative data. Problematically, this approach has led to the RNA world model being populated with RNAs whose distributions are patchy, and antiquity has often been inferred on speculative grounds, following detailed experimental characterisation of RNAs from a handful of model organisms. Against this backdrop, it is perhaps of little surprise that more vociferous critics have dubbed this endeavour the ‘RNA dreamtime’ [33].

While detailed studies have been performed for single RNA families (Table S1 in Text S1), no published data present a systematic analysis covering all RNA families, despite this now being routine for protein-coding genes. For RNA genes, an equivalent analysis is long overdue but has not been possible because, until recently, comparative data were not of sufficiently high quality.

We therefore sought to systematically address whether the phylogenetic distribution of extant RNAs fits with direct descent from an RNA world, as predicted under the Continuity hypothesis, or whether the distribution of extant RNAs better reflects more recent (post-LUCA) origins. In addition, we sought to examine whether horizontal transfer between cellular domains (and viruses) is detectable for RNA families. We report an analysis of over 3 million RNAs spanning 1446 families in the Rfam database [34], revealing that the overwhelming majority of families (99%) are restricted to a single domain of life. By contrast, fewer than 1% show evidence of either a deeper evolutionary origin, or of interdomain transfers. We conclude that, while, on these proportions, the RNA world ‘palimpsest’ is only a fraction of the RNA repertoires of modern genomes, the most ancient RNA families nevertheless belie evidence of an advanced protein synthesis apparatus. Strikingly, we report that interdomain horizontal gene transfers are also minimal for RNA genes, in marked contrast to the significant levels detected for protein-coding genes. Our analyses thus serve to move the current state-of-the-art from erudite literature review to systematic analysis of the distribution and antiquity of large numbers of RNA families.

## Results/Discussion

### 99% of RNA families are restricted to a single domain of life

We first asked whether a systematic analysis of RNA families expands our knowledge of ancient RNAs beyond those identified by traditional experimental work. To examine the degree to which extant RNAs can be traced to earlier evolutionary periods, we performed comparative analyses of annotated RNAs based on data from all three domains of life as well as viruses. To this end, we used the Rfam (RNA families) database [34], which groups RNAs into families, and families into clans, based on manually-curated alignments, consensus secondary structures, covariance models [35] and functional annotations. RNAs within families and clans can therefore be claimed to share a common ancestry [34]. All analyses presented here are based on Rfam 10.0, which consists of over 3 million annotations grouped into 1446 families and 99 clans [34].

**Figure 1:**
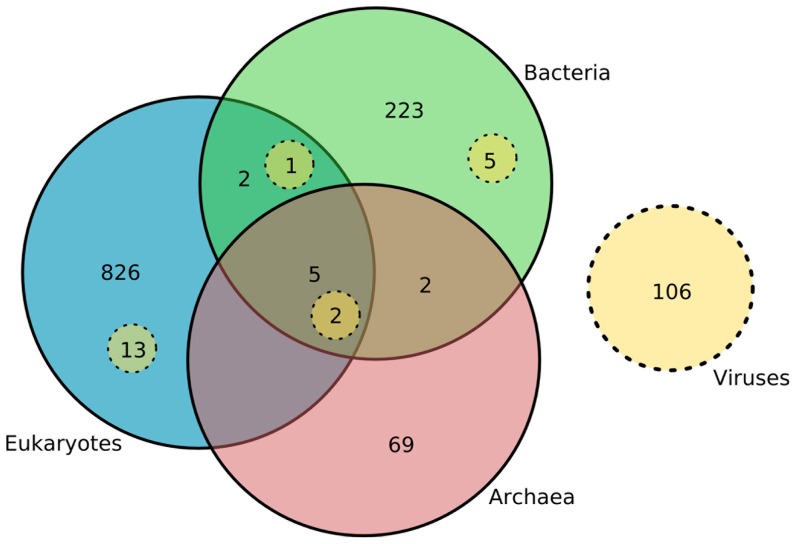
Venn diagram of RNA family distribution. Taxonomic information attached to EMBL-derived Rfam annotations reveals that the majority (99%) of RNA families are domain-specific, with only seven RNA families universally conserved (across the three domains of life plus viruses; Table S1 in Text S1). Numbers within dashed circles indicate viral RNA families.

To generate a high-quality dataset, we first established the distribution of all individual RNA sequence entries in Rfam by reference to the NCBI taxonomy database, and manually vetted and removed probable false positive annotations. From the resulting dataset, we generated an initial survey of families and clans across bacterial, archaeal, eukaryotic and viral genomes (**Figure 1**). Two patterns are immediately clear. First, each domain carries a large number of entries absent from the other domains, with limited overlap observed between domains, or with viruses. Second, only seven Rfam families are present across all three domains. That we observe distinct domain-level RNA repertoires appears consistent with the view that the three domains of life are genetically distinct [36]. However, families present in more than one domain (or shared with viruses) may be the result of either vertical evolution from a common ancestor or horizontal transfer of genes between domains [30], [36].

### Interdomain RNA families show a mix of vertical and horizontal inheritance

**Figure 2:**
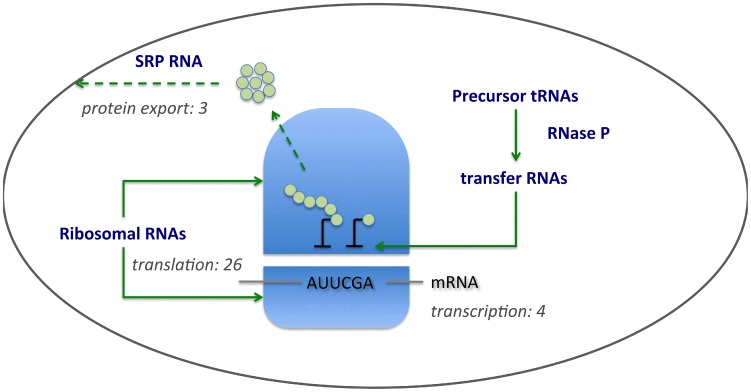
RNA-based processes traceable to the Last Universal Common Ancestor. Universal Rfam families that show evidence of vertical inheritance (Table S1 in Text S1) are all associated with the processes of translation (rRNAs, tRNAs, RNase P) and protein export (SRP RNA). A previous study examining the antiquity of protein coding genes [21] identified only 37 universally distributed proteins which show evidence of vertical inheritance. The majority of these vertically inherited proteins are associated with translation and protein export; numbers of such proteins associated with each of the depicted processes is given in grey (original data are from Harris [21]). The proteins associated with RNase P are not universally conserved, with archaeal and eukaryotic RNase P proteins being unrelated to their bacterial counterparts [72]. While tRNA synthetases are universal, they have undergone ancient horizontal gene transfer events [73], which complicates establishing the timing of their origin.

We next sought to establish whether the distribution the 12 interdomain Rfam families/clans (**Figure 1**) could be attributed either to vertical inheritance or horizontal gene transfer. Previous studies and data on distribution allow a predominantly vertical pattern of inheritance to be attributed to only five families (small subunit (SSU) and 5S rRNAs, tRNA, RNase P RNA, signal recognition particle RNA (SRP RNA) with four showing evidence of HGT (group I & II introns, organellar large subunit (LSU) rRNA, IsrR RNA) (Table S1 in Text S1). Ribosomal RNAs are not fully represented in Rfam, being amply covered by other databases (e.g. [37], [38]), but their deep evolutionary history has been readily traced (Table S1 in Text S1). Combined, these data confirm a minimal reconstruction of the RNA repertoire of LUCA consistent with that observed for protein-coding genes [21], with the demonstrably oldest RNAs and the majority of such proteins being involved in translation and protein export (**Figure 2**). Consequently, while the number of RNA families traceable to LUCA is an order of magnitude lower than for proteins, the spread of functionalities is nevertheless very similar in extent.

**Figure 3:**
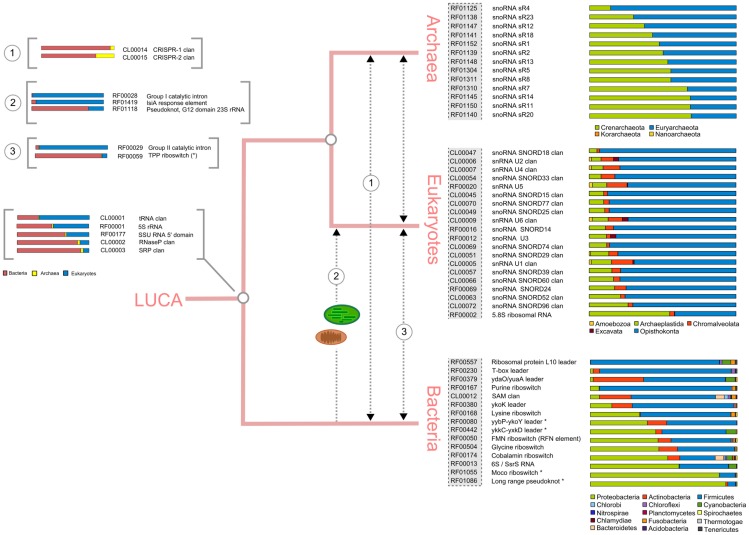
Reconstruction of broadly distributed RNA repertoires for each domain, plus interdomain RNA families. Colored bars at far right indicate normalized taxonomic abundance of each Rfam for major taxonomic groupings within each domain. Horizontal traces (see text, Table S1 in Text S1) for interdomain families, are depicted as follows: general transfer patterns are given by dashed arrows; proposed HGT patterns for individual families are depicted by number (inset). For Rfam families present in more than one domain (far left and inset), bars indicate normalized taxonomic abundance by domain (color scheme at bottom left). Asterisks indicate additional broadly-distributed bacterial candidates identified using GEBA tree topology [56] (see text). Note that the Rfam rRNA families in Rfam 10.0 are based on conserved subsequences, and are not as comprehensive as other resources (see main text) and are included here for consistency. The universally-distributed rRNAs are the small subunit (16/18S) rRNA, large subunit (23/28S) rRNA and 5S rRNA (see Table S1 in Text S1). The 5.8S rRNA of eukaryotes is known to be homologous to the 5′ end of bacterial and archaeal 23S rRNA [74], [75], so its inclusion as a eukaryote-specific family in Rfam is in this respect artefactual.

A vertical trace is suspected but not demonstrated for the universally distributed TPP riboswitch (Table S1 in Text S1, **Figure 3**), which modulates gene expression in response to thiamine pyrophosphate (TPP). The analysis of patterns of inheritance for RNAs is complicated by their short lengths and generally low levels of sequence conservation. As riboswitches regulate cognate mRNA in cis, vertical transmission may be tested by generating phylogenies from the protein products, on the assumption that the riboswitch and ORF have coevolved. We therefore generated a phylogeny for THIC, the only TPP-regulated gene product present in all three domains. The phylogeny shows eukaryote sequences grouping with proteobacteria (**Figure S1**), consistent with horizontal transmission of TPP-riboswitch regulated ThiC to the eukaryote lineage from a bacterial donor. Several independent observations are consistent with horizontal transmission: *Arabidopsis* THIC is nuclear-encoded, but targets to the chloroplast [39], plant ThiC can complement an *E. coli* ThiC mutant [40], and eukaryotic TPP riboswitches show limited distribution [41] (Rfam 10.0). Moreover, THI1, which also carries a TPP riboswitch in its mRNA leader, is also targeted to chloroplasts and mitochondria [42]. While an early origin for TPP riboswitches [11] remains plausible, this is difficult to reconcile with our THIC phylogeny, since bacterial and archaeal sequences are not monophyletic under any rooting (**Figure S1**).

Also noteworthy is the CRISPR/Cas system, which combats viral and plasmid infection in both bacteria and archaea. Horizontal transmission has been suggested for this system, but interdomain transfer is thought to be limited [43]. Examination of CRISPR crRNA family distribution reveals that 54 of 65 Rfam crRNA families are restricted to a single domain (Table S2 in Text S1). The remaining 11 families fall into two clans (CRISPR-1, CRISPR-2), which include crRNAs in both bacterial and archaeal genomes. However, only one Rfam family from each of these two clans contains annotations deriving from both domains. While short sequence length of crRNAs precludes phylogenetic analyses, the distribution we report (Table S2 in Text S1) is compatible with sporadic interdomain transfer, consistent with a phylogenomic analysis of Cas genes/clusters which reported low levels of horizontal transmission [44].

The low number of observed interdomain RNA families suggests that, in contrast to protein-coding gene repertoires, RNA repertoires are surprisingly refractory to interdomain transfers. While we do see evidence of organellar contributions, these are few in number, in marked contrast to the high numbers observed for protein-coding genes [45], [46].

### Only a minority of domain-specific RNA families are broadly-distributed

We next sought to establish the distribution of RNA families within each domain, since our initial analysis (**Figure 1**) does not consider within-domain taxonomic distribution of Rfam families. A broad distribution may indicate an early origin of a given family, but information on distribution alone cannot distinguish between horizontal and vertical modes of transmission. As short length and limited sequence conservation preclude robust phylogenies for the vast majority of RNA families, distribution cannot be used to directly infer the RNA repertoire of the last common ancestor (LCA) of each domain. Nevertheless, such information may indicate whether the RNA repertoires of the three domains are functionally distinct. We therefore collated families present in at least 50% of major within-domain taxonomic divisions (**Figure 3**, **Dataset S2**). Surprisingly, the number of broadly distributed families/clans within each domain is small (Archaea 13/69 = 18.8%, Bacteria 15/223 = 6.7%, Eukaryotes 20/826 = 2.4%), though among eukaryotes there are a high number of clans, which may encompass multiple RNA families with a shared evolutionary history. Two patterns emerge from this analysis (**Figure 3**). First, eukaryote and archaeal repertoires are dominated by small nucleolar RNAs (snoRNAs). Second, the most broadly distributed bacterial RNAs are regulatory.

Closer investigation of the snoRNA repertoires across archaea and eukaryotes reveals that C/D family RNAs are broadly distributed; H/ACA family RNAs, while widespread among eukaryotes, are only known from Euryarchaeota [47], [48], and Archaeal H/ACA RNAs are not currently included in Rfam [34]. Strikingly, of the >500 snoRNA families included in this study, none are shared across archaea and eukaryotes. While a deep origin of snoRNPs is supported by surveys of protein and RNA components [49], this is not reflected by existence of conserved RNA families [28], for which only scant evidence exists [50], [51].

In eukaryotes, a strong domain-specific evolutionary trace is attributable to snRNAs (**Figure 3**, Table S3 in Text S1), consistent with other studies indicating both the major and minor spliceosome were features of the Last Eukaryotic Common Ancestor (LECA) [52]–[54].

A different picture emerges for miRNAs however. The broad distribution of miRNAs is consistent with the suggestion that RNAi pathways trace to the LECA [55], with 26/452 miRNA families present in more than one eukaryotic supergroup (**Dataset S3**). However, closer inspection reveals most are singleton false positives or artefactual family groupings. Our dataset therefore does not allow the placement of any individual miRNA families in LECA.

**Figure 4:**
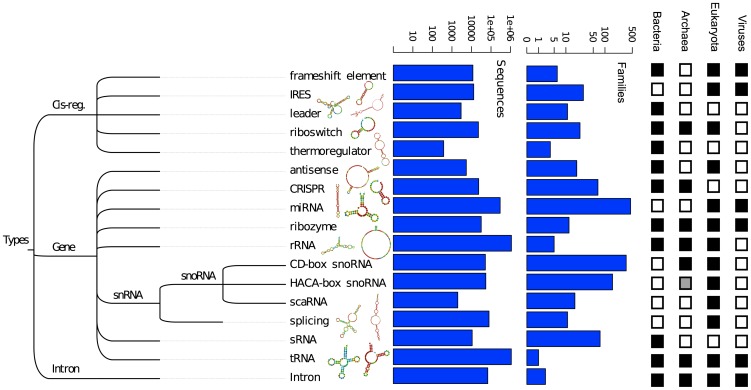
Rfam-based functional classification of RNA families. The tree depicts classification of the higher level data structures within Rfam, and is not a phylogeny. Numbers of sequences and families in Rfam 10 that fall into each functional classification are shown as bar charts. Domain-level taxonomic distribution for each functional category is shown by black (present) and white (absent) boxes, right. The grey box indicates that H/ACA family RNAs are known from archaea [47], [48], but are not in Rfam 10.

A broad qualitative difference between bacteria compared to archaea and eukaryotes is the preponderance of conserved regulatory elements, primarily riboswitches (**Figure 3**). However, this observation is based on only that small fraction of Rfam families present in ≥50% of taxonomic divisions. To further assess whether there are qualitative differences between the functional RNA repertoires across the three domains and viruses, we took advantage of the organization of Rfam into different functionalities. As is evident from **Figure 4**, common functionalities across all three domains are sparse. Riboswitches and ribozymes indicate the ubiquity of small metabolite-based regulation and catalytic function, but of the numerous families included in this analysis, only RNase P RNA is directly traceable to the LUCA (**Figures 2** **&** **3**). Functionalities shared between archaea and eukaryotes to the exclusion of bacteria are restricted to snoRNA-dependent RNA modification, and CRISPRs are the only prokaryote-specific functionality. Interestingly, a number of RNA functionalities present in bacteria lack archaeal or eukaryotic representatives (cis-regulatory leaders, thermoregulators, sRNAs), and Rfam contains no archaeal-specific functionalities (**Figure 4** **, Dataset S4**), possibly attributable to the smaller number of experimental screens for novel RNAs across members of this domain.

### Biases in taxonomic sampling

In comparing the RNA repertoires of the three domains, a key question is whether the underlying Rfam data cover a reasonable spread of species within each domain, or whether data from a few species or phyla dominate. This is important in that the low number of broadly distributed families/clans we observe within each domain could be the result of an underlying sampling bias. A priori we may expect a significant bias, given current genomic coverage of microbial biodiversity. For instance, a recent survey of snoRNAs indicates there is broad, though nevertheless patchy coverage across major eukaryotic and archaeal groups [49]. We therefore examined the underlying taxonomic distribution of all domain-specific Rfams. For all three domains, entries are heavily skewed, with a majority of Rfam annotations deriving from a narrow phylogenetic diversity (**Figure S2**).

For protein-coding genes, discovery of novel proteins has been significantly enhanced by sequencing of genomes chosen for maximal phylogenetic diversity [56]. While de novo computational discovery of novel ncRNAs is non-trivial by comparison, we were nevertheless interested in establishing whether the additional phylogenetic coverage provided by the Genomic Encyclopedia of Bacteria and Archaea (GEBA) [56] impacted the number of broadly distributed Rfam families. Under the assumption of vertical inheritance, we therefore treated RNAs as characters on the GEBA phylogeny. Our analysis yielded four additional bacterial candidates (marked with asterisks in **Figure 3**), though again we caution that broad distribution may be generated through HGT, so these candidates cannot be placed in the bacterial ancestor. Nevertheless, this modest improvement suggests GEBA [56], and targeted experimental screens informed by phylogeny [49] will provide a valuable framework, both for improving knowledge of RNA family distribution and in focusing experimental screens for novel RNA families.

How should we interpret these data? The limited distribution of domain-specific RNAs is likely to be biased by sampling, a problem that affects all genomic data, and is even more acute for detailed experimental data. On available data, we find that only a minority of domain-specific RNAs exhibit a broad distribution. A broad distribution could result from vertical inheritance, but it could also be the result of horizontal gene transfer. Taxonomic biases might underestimate the number of RNAs vertically traceable to the ancestor of a domain, whereas horizontal gene transfer might be expected to expand the distribution of some RNAs. Assuming that current sampling has gaps, but is not completely uninformative [49], available data suggest that a high proportion of RNAs are likely to be evolutionarily young, and will not trace to the LCA of the domain in which they reside.

### Concluding remarks

We have examined the evolution and diversity of RNAs across the entire tree of life, an important complement to previous comparative studies on RNA metabolism [11], [17] and RNA-associated protein families [57]. Large-scale analyses of the RNA repertoire are only now becoming possible through improved methodologies for RNA identification and greater integration between RNA discovery and online databases.

It is commonplace for novel RNAs or RNA families to be discussed in regard to their potential relevance to the RNA world, yet RNAs with limited distribution are difficult to reconcile with a very ancient evolutionary origin unless massive losses are invoked. Excepting the possibility of losses (which cannot be readily tested since the evidence for antiquity has been erased), our study shows that direct evidence for the RNA continuity hypothesis remains scant; there is undoubtedly an RNA ‘palimpsest’ [16], but it is not possible to expand this through systematic comparative analyses.

Conversely, we find clear evidence of distinct domain-level repertoires, but limited evidence of inter-domain transfers, consistent with a recent analysis indicating a detectable vertical trace amidst ongoing HGT [30]. The paucity of shared eukaryotic and archaeal RNA regulatory processes (**Figure 4**) and the marginal bacterial contribution to the eukaryote RNA repertoire, support the view that eukaryotic mechanisms of RNA regulation are a domain-specific invention [15], and extend this view to the other two domains. While we see qualitative similarities between archaea and eukaryotes (**Figures 3** **&** **4**), in agreement with studies indicating a phylogenetic affinity between these two domains [58], these are currently restricted to snoRNAs. The clear differences in RNA functional repertoires between eukaryotes, archaea and bacteria (**Figure 4**) strengthen the case for recognizing the *biological* distinctness of the three domains [36], independent of uncertainty surrounding their specific phylogenetic relationships [59].

## Materials and Methods

### Rfam dataset

Annotated noncoding RNA data used in this study was derived from data curated in Release 10.0 of the Rfam database [34] (http://rfam.sanger.ac.uk/). The distribution of Rfam families (**Dataset S1**) was established in two steps. First, for a given family, all annotations across the EMBL database [60] (http://www.ebi.ac.uk/embl/) were binned into domains using the taxonomic information attached to each sequence. We then inspected annotations from families whose distribution spanned more than one domain to identify possible false annotations. For all Rfam families with annotations spanning two or more domains (including viruses) we first confirmed the taxonomic affiliation of each sequence through reciprocal blasts against the GenBank database and removed any cases where sequences were clearly misannotated (e.g. bacterial sequencing vectors in eukaryote genome projects). Next, we inspected the quality of each annotation with reference to Rfam seed alignments. Any sequences with a bitscore within +10 bits of the individual bitscore cutoffs for curated seed alignments, and where sequence similarity was deemed insufficient to reliably establish homology, were discarded.

### Higher-level taxonomic assignments

In assigning Rfam entries to specific taxonomic groups of bacteria and archaea (**Figure 3** **, Dataset S1**), we used the top-level classifications within each domain in the NCBI Taxonomy Database. At the time the analyses were performed, the proposed archaeal phylum Thaumarchaeota [61] was not recognised in the database, and available sequences were classified as Crenarchaeota. While members of the Thaumarchaeota are present in our data, none carry annotated snoRNAs, so not explicitly recognizing putative Thaumarchaeotes as a phylum does not impact the results summarized in figure 2. For Eukaryote RNA sequences, data was grouped according to the classification scheme proposed by Adl and colleagues [62].

### Phylogenetic analyses

All sequences annotated as THIC in Genbank were retrieved (8 Feb 2011). The resulting list of 4508 sequences were examined for sequence similarity by generating a blast network using the blastall program from the BLAST package (version 2.2.18), with an E-value cutoff of 0.1. The network of blast results was visualized with CLANS [63], using default settings. The output was then clustered using MCL [64], with granularity set at 4. Representative sequences spanning all domains were retrieved from all MCL clusters with >10 members. Sequences were aligned using MSA-Probs [65]. Partial sequences and extremely divergent sequences where annotation appeared questionable were removed. Conserved regions were selected for use in phylogenetic analysis via the G-blocks server [66] (http://molevol.cmima.csic.es/castresana/Gblocks_server.html), with the settings ‘Allow smaller final blocks’ and ‘Allow gap positions within the final blocks’ selected. ProtTest [67] was used to identify the best-fit model of protein evolution for our alignment. Phylogenetic analysis was performed using PhyML 3.0 [68] with parameters and model (WAG+I+G) as selected using ProtTest. Bootstrapping was performed on two Mac Pro machines with Intel Xeon Quad core processors, running 12 parallel threads. Parallelization yielded a total of 108 bootstrap replicates (a consequence of running 12 threads in parallel, resulting in bootstrap replicates that were a multiple of 12); all bootstrap values in figure S1 are therefore out of a total of 108 not 100. Additional trees were generated using RAxML [69] and BioNJ [70] to assess robustness of the topology. Tree figures were generated in Dendroscope [71].

## Supporting Information

Dataset S1 **Distribution of Rfam families across domains and major phylogenetic groups.**
(XLS)

Dataset S2 **Distribution of archaeal, eukaryote and bacterial Rfams.**
(XLS)

Dataset S3 **Distribution of eukaryotic miRNAs in Rfam.**
(XLS)

Dataset S4 **Numbers and taxonomic sources of annotations associated with RNA functional groups.**
(XLS)

Figure S1 **Unrooted PhyML phylogeny of TPP-regulated gene product THIC.** (A) Tree in landscape format so labels are legible. The phylogeny shows good support for a close affinity between Plant and green algal (green) and a clan of proteobacterial homologs (red), to the exclusion of archaeal sequences (dark blue), consistent with possible HGT from bacteria to eukaryotes. Monophyletic groups are not recovered for either archaea or bacteria, suggestive of horizontal transmission events. All tips are labeled with the following information: MCL_cluster|Domain|gi_number|species_name. Bootstrap values are out of 108 (Materials and Methods). (B) Same tree in unrooted form; coloring is identical to key in (A).
(TIF)

Figure S2 **Analysis of taxonomic distribution of Rfam entries within the EMBL nucleotide database.** Data for each of the three domains (A) Eukarya (B) Archaea (C) Bacteria are binned by indicated major taxonomic groupings (see Materials and Methods). The x-axis corresponds to individual Rfam entries. The majority of families are restricted to well-studied groups, revealing a strong bias in the underlying data, as previously seen for snoRNA families [49] and more generally for genome projects [56].
(TIF)

Figure S3 **Discovery curves for Rfam.** These curves plot the oldest reliable electronic date (EMBL entry or publication) associated with a particular Rfam family. Domain distribution (1-domain, 2-domain or 3-domain) is based on current distributions. To generate discovery curves for all RNA families in Rfam 10.0 (which includes families built before January 2010), we extracted the oldest dates from the literature references contained in the corresponding Stockholm file and from the EMBL accessions – the oldest date of the two is plotted.
(TIF)

Text S1 **PDF with supporting text and references, and supplementary tables S1–S4.**
(DOC)
