# Supplementary figures and images for: Comparative Analysis of RNA Families Reveals Distinct Repertoires for Each Domain of Life
Source: PLoS Comput Biol. 2012 Nov 1;8(11):e1002752. doi: 10.1371/journal.pcbi.1002752 (PMC3486863; doi:10.1371/journal.pcbi.1002752)

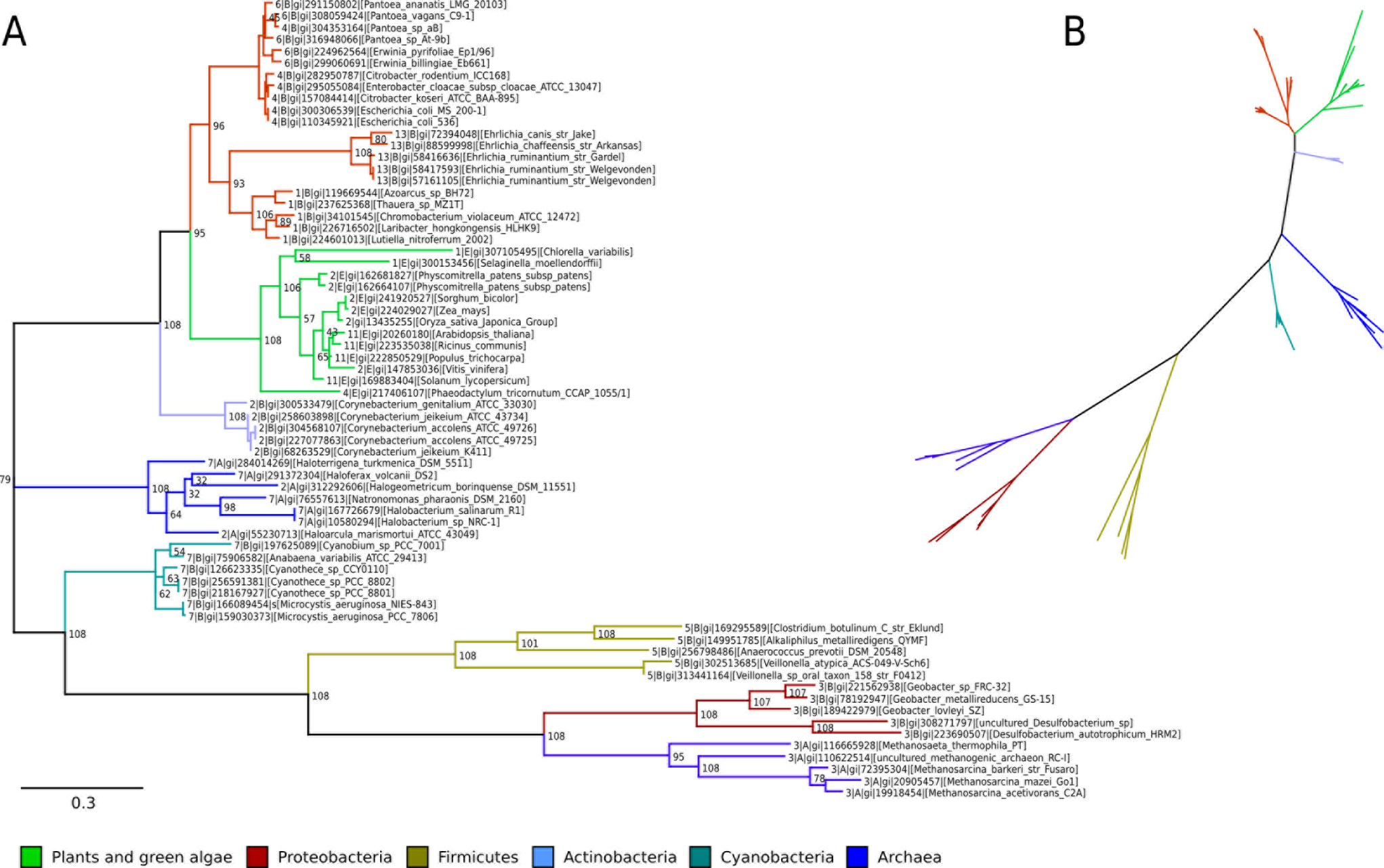

Supplement: Figure S1 — Unrooted PhyML phylogeny of TPP-regulated gene product THIC. (A) Tree in landscape format so labels are legible. The phylogeny shows good support for a close affinity between Plant and green algal (green) and a clan of proteobacterial homologs (red), to the exclusion of archaeal sequences (dark blue), consistent with possible HGT from bacteria to eukaryotes. Monophyletic groups are not recovered for either archaea or bacteria, suggestive of horizontal transmission events. All tips are labeled with the following information: MCL_cluster|Domain|gi_number|species_name. Bootstrap values are out of 108 (Materials and Methods). (B) Same tree in unrooted form; coloring is identical to key in (A). (TIF) [file pcbi.1002752.s005.tif]

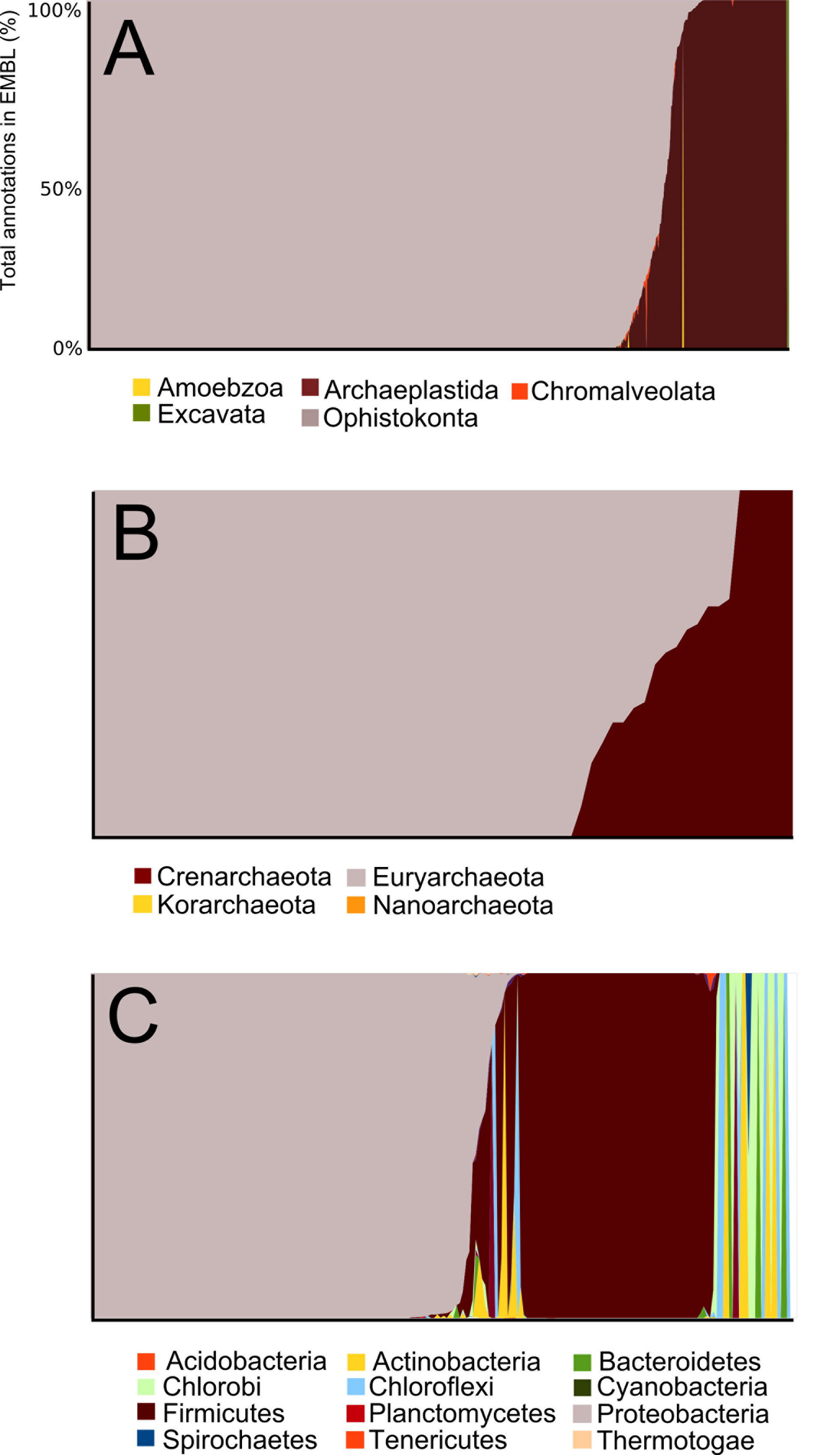

Supplement: Figure S2 — Analysis of taxonomic distribution of Rfam entries within the EMBL nucleotide database. Data for each of the three domains (A) Eukarya (B) Archaea (C) Bacteria are binned by indicated major taxonomic groupings (see Materials and Methods). The x-axis corresponds to individual Rfam entries. The majority of families are restricted to well-studied groups, revealing a strong bias in the underlying data, as previously seen for snoRNA families [49] and more generally for genome projects [56]. (TIF) [file pcbi.1002752.s006.tif]

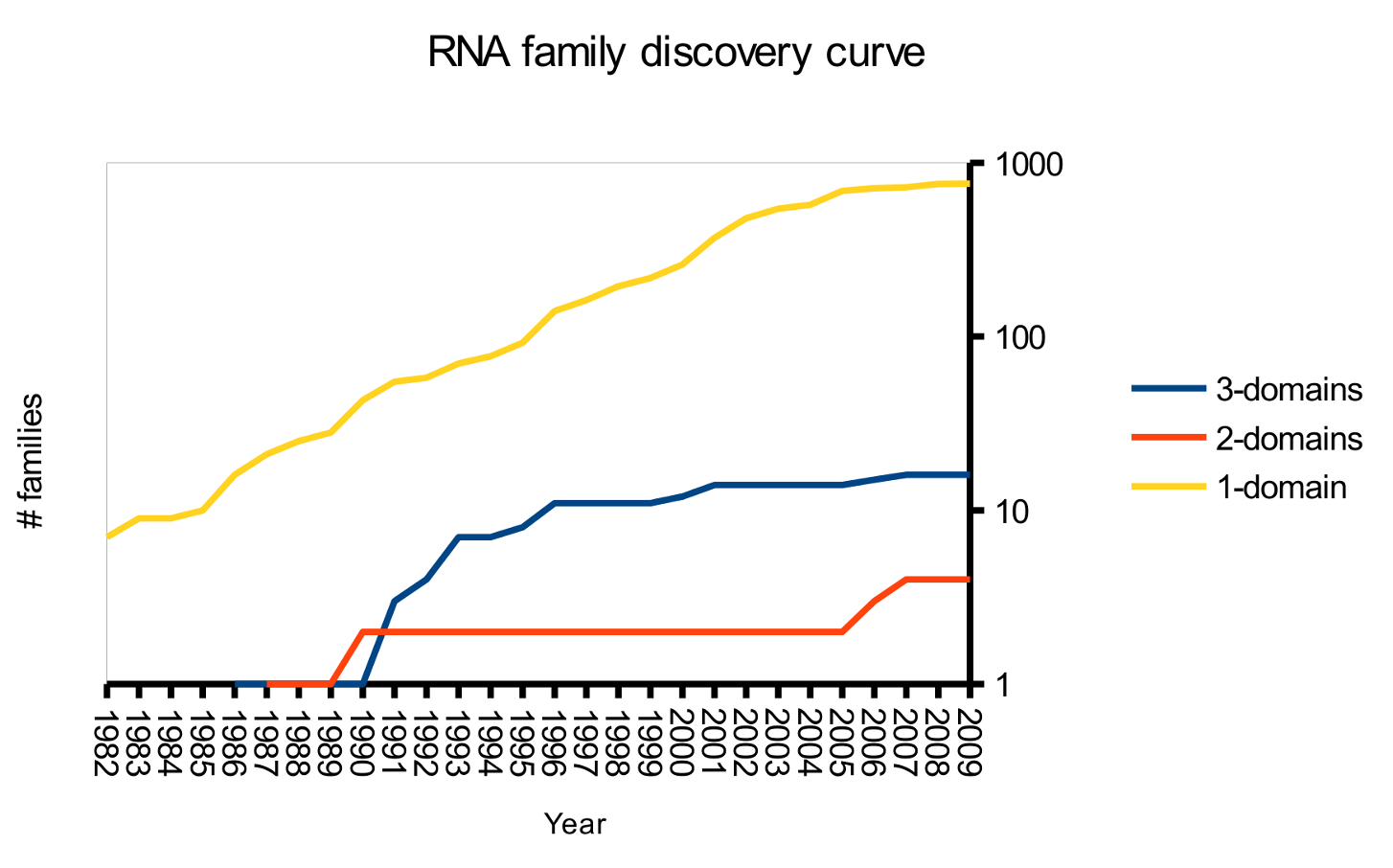

Supplement: Figure S3 — Discovery curves for Rfam. These curves plot the oldest reliable electronic date (EMBL entry or publication) associated with a particular Rfam family. Domain distribution (1-domain, 2-domain or 3-domain) is based on current distributions. To generate discovery curves for all RNA families in Rfam 10.0 (which includes families built before January 2010), we extracted the oldest dates from the literature references contained in the corresponding Stockholm file and from the EMBL accessions – the oldest date of the two is plotted. (TIF) [file pcbi.1002752.s007.tif]
