# Supplementary material for: Comparative Analysis of RNA Families Reveals Distinct Repertoires for Each Domain of Life
Source: PLoS Comput Biol. 2012 Nov 1;8(11):e1002752. doi: 10.1371/journal.pcbi.1002752 (PMC3486863; doi:10.1371/journal.pcbi.1002752)
Supplement: Text S1 — PDF with supporting text and references, and supplementary tables S1–S4. (DOC) [file pcbi.1002752.s008.doc]

Supporting Information for

**Comparative analysis of RNA families reveals distinct repertoires for each domain of life**

Marc P. Hoeppner^a^, Paul P. Gardner^b^ & Anthony M. Poole^b^*

^a^Science for Life Laboratory, Department of Medical Biochemistry and Microbiology, Uppsala University, SE-751 23 Uppsala, Sweden

^b^Biomolecular Interaction Centre & School of Biological Sciences, University of Canterbury, Private Bag 4800, Christchurch 8140, New Zealand

*To whom correspondence should be addressed.

Email addresses:

anthony.poole@canterbury.ac.nz

paul.gardner@canterbury.ac.nz

mphoeppner@gmail.com

**Supporting Information**

*Justification for the use of Rfam data for comparative analyses.*

There are several issues that must be considered for any analysis that requires homology inference. As a large scale comparative analysis of RNA families has not, to our knowledge, been performed before, it is important to begin such an analysis with a discussion of the merits — and possible limitations — of analyzing such a dataset across deep evolutionary history.

For both proteins and RNA, structure is often better conserved than sequence, such that homology may not be detectable from sequence data alone . In Rfam, families are based on covariance models (CMs), which contain both primary sequence and secondary structure information , thus enabling detection of homology well below the twilight zone of sequence similarity for nucleic acids . Evolutionary relationships for RNA genes from distant taxa have been reported (**Table S1**), and such distant similarities can be detected using Rfam , suggesting detection of homology is possible with a range of methods, even for distantly related RNAs.

Major classification schemes for protein families are based on measures of sequence similarity , which may fracture protein families classified from structure, where significant sequence similarity is undetectable (e.g. ribonucleotide reductases — ). If homology is routinely missed for deeply conserved RNAs, biologically-characterisable families should be artificially fractured. Examination of Rfam revealed few obvious cases, with those that we could identify being resolved at the clan level . In Rfam 10.0, 20% of families are further grouped into clans, and clan generation is achieved via implementation of a modified version of PRC , optimized for RNA profile:profile comparisions . This permits detection of very distant relationships, based on both sequence and secondary structure similarity across multiple Rfam families. While no homology detection method can be claimed to be exhaustive, we believe Rfam is, in methodological terms, comparable to best practice in delineation of protein families by profile:profile comparisons . For both types of data (RNA and protein), homology inference (i.e. defining families as a collection of sequences with a shared common ancestor) is made based on a measure of similarity. There are known issues with existing sequence databases and datasets, where distinguishing between different forms of homology may be non-trivial, and is often not explicitly established . As with protein families, Rfam families may therefore contain a mixture of orthologs (genes related by common descent following speciation), paralogs (genes related by common descent following gene duplication) and xenologs (genes related by common descent following horizontal gene transfer). For xenologs, this will not obviously affect the number of Rfam families, only their distribution. As discussed below, since this study focuses only on interdomain comparisons, we explicity examine xenologs at the level of interdomain comparisons, but not within domains. Within-domain xenology may impact the number of broadly-distributed RNAs. More generally, at the level of resolution used here (comparison across the three domains of life), failure to distinguish between orthologs and paralogs may at most alter the number of families attributed to each domain. Cases where paralogs are counted as separate families would artificially increase the number of within-domain families, and cases where functionally divergent paralogs are grouped within the same family would reduce this number. Mitigating against this, Rfam families and clans are based on a combination of sequence and structural similarity, plus common functionality , and inspection of clans indicates these represent orthologous groups rather than groupings of larger families with multiple paralogous constituents (personal observations). We think it is reasonable to conclude that the RNAs that make up individual Rfam families and clans can be considered to be homologous, and duly note that the caveats described here regarding orthology and paralogy apply equally to large protein-based datasets . We can identify no sources of error that are demonstrably associated only with RNA data. For these reasons, we conclude that Rfam data is amenable to global comparative analyses.

*Rates of interdomain RNA family discovery.*

Given the currently rapid rate of discovery of novel RNAs, the Rfam database may not carry an up-to-the-minute picture of all known RNA families. The analysis we present is therefore necessarily a snapshot of current knowledge at the time of the Rfam release on which it is based, and will no doubt evolve as new RNA families are discovered. For the current study it is important to establish the rate of discovery of interdomain RNAs relative to intradomain RNAs. We therefore plotted discovery curves for all of Rfam (**Figure S3**). As is clear from **Figure S3**, the discovery rate of interdomain RNAs flattened off some time ago, whereas even in a conservative database like Rfam, domain-specific RNAs are still being added at a significant rate. There is no indication that interdomain RNAs lag far behind in terms of discovery. As is clear from **Figure S3** and **Table S4**, some newer cases even show a shortening of discovery times, perhaps because it is easier to screen for these in the post-genomic environment (e.g. ). We suspect that, as new data are published (as discussed in e.g. ), single-domain RNA families will continue to massively outpace discovery of new interdomain RNAs.

*Universally-distributed RNA families.*

Two families/clans show a universal distribution (present in all three domains plus viruses). For group II self-splicing introns, it is well established that these RNA elements are horizontally transmitted, with good evidence for recent transfer events from bacteria to archaea , and to eukaryotes via organelles . By contrast, tRNAs, which are also universal, have been proposed to show a vertical evolutionary trace , and their involvement in viral replication has been argued to indicate an early evolutionary origin . While individual tRNAs may have polyphyletic origins , placing presence of this family of RNAs in the ancestor of all three domains (**Figure 2**) is not controversial.

*RNA families present in all three domains.*

Five RNA families/clans are present in all three domains, and four of the five have been previously argued to show a vertical trace (**Table S1**). Rfam does not include full models for the large and small subunit ribosomal RNA, though RF00177 covers the 5' domain of the SSU rRNA. The only surprise member of this list is the TPP riboswitch. A difficulty with directly examining the evolutionary history of specific RNA elements in detail is that elements tend to be short, precluding reliable phylogenetic analysis in many cases. To abrogate this problem, we generated protein sequence phylogenies (Materials and Methods) derived from the most broadly distributed TPP-regulated gene, ThiC. With in excess of 4500 THIC sequences in genbank, we used MCL to generate a broad overview of the data and selected representative sequences from each of the major MCL clusters for phylogenetic analysis (see Materials and Methods). Next, we performed phylogenetic analyses on a subset of this data, with sequence selection guided by the network of clusters (Materials and Methods). As is clear from **Figure S1**, we do not recover the monophyly of the three domains, with proteobacteria and archaea both split into distinct groups, which cannot be attributed to phylogenetic artefact. It therefore seems likely that the non-monophyly of both archaeal and bacterial ThiC sequences is best attributed to horizontal transmission events. Eukaryotes, in contrast, do form a single clan (sensu ). While the tree in **Figure S1** is unrooted, vertical descent of the eukaryote sequences from the Last Universal Common Ancestor is difficult to reconcile with the non-monophyly among the other two domains. This would require the position of the root to be between eukaryotes and bacteria/archaea. Given that the eukaryote sequences group with proteobacterial sequences, are relatively restricted in distribution, and surrounded by neighbouring bacterial clans, it seems more plausible that eukaryote ThiC sequences have entered this domain via horizontal gene transfer from a bacterial source during the evolution of the Archaeplastida.

*Interdomain RNA families.*

After vetting for false annotations, we recovered five additional families/clans with members present in more than one domain (**Table S1**). Evidence of horizontal transmission can be established for all five cases. **Table S2** shows that the distribution of CRISPR crRNA Rfam families is largely domain-specific, suggesting ongoing interdomain transfer is minimal. 53 of 65 families we analyzed are present in only a single domain. One family (RF01353) contains both archaeal and bacterial sequences but closer inspection reveals it to be archaeal-specific; this family carries only a single bacterial annotation, in Cyanothece sp. CCY0110, based on a single non-repeated, non-Cas associated region, making this almost certainly a false annotation. As noted in the main text, only two families show interdomain distribution, indicating at most limited interdomain transfer of crRNAs.

As per group II introns above, the horizontal transmission of group I self-splicing introns is well-documented (**Table S1**; ). The LSU rRNA pseudoknot is present in 23S rRNA from bacteria and eukaryotic organellar 23S rRNA; the latter entered eukaryotes via bacterial endosymbioses, as judged by representative 23S rRNA phylogenies, and congruence with 16S rRNA phylogenies . All eukaryotic group II intron and 23S rRNA sequences annotated in the EMBL database were examined to establish their genomic location; in all cases, we find these are in organellar (chloroplast and mitochondrial) genomes. Finally, the IsrR iron stress repressed RNA is associated with photosystem I in the cyanobacterium *Synechocystis* sp. PCC 6803 , and annotated eukaryote sequences in the EMBL database are all chloroplast-encoded, strongly linking this element to the endosymbiotic origin of the chloroplast.

**Table S1. Conservation of Rfam families and clans across domains & viruses**

| **Distribution** | **RNA** | **Rfam ID** | **Evolutionary trace** |
| --- | --- | --- | --- |
| **Universal** | tRNA | CL00001 | Vertical |
|  | Group II intron | RF00029 | Horizontal: Bacteria to Archaea ; Bacteria to Eukaryotes via organelles ; examination of taxonomic distribution of eukaryotic group II introns annotated in EMBL (this study; see also <http://www.rna.ccbb.utexas.edu/SAE/2C/>) confirms all are encoded in chloroplast and mitochondrial genomes. |
| **3 domains** | Large and small subunit RNA | N/A | Vertical |
|  | SSU RNA, 5' domain | RF00177 | Vertical |
|  | 5S rRNA | RF00001 | Vertical |
|  | TPP riboswitch | RF00059 | Horizontal: this study (Figure S2). |
|  | RNase P RNA | CL00002 | Vertical |
|  | SRP RNA | CL00003 | Vertical |
| **Prokaryotes** | crRNA: CRISPR-1 | CL00014 | Horizontal |
|  | crRNA: CRISPR-2 | CL00015 | Horizontal (as above) |
| **Viruses, Bacteria & Eukarya** | Group I intron | RF00028 | Horizontal: Bacteria to Eukaryotes via organelles ; examination of taxonomic distribution of eukaryotic group I introns annotated in EMBL (this study) confirms all are encoded in chloroplast and mitochondrial genomes. Note however that group I intron insertion into nuclear rRNA genes has also been described ; <http://www.rna.ccbb.utexas.edu/SAE/2C/>) |
| **Bacteria & Eukarya** | 23S rRNA Domain G (G12) pseudoknot | RF01118 | Vertical & horizontal: bacteria to eukaryotes ; examination of taxonomic distribution of eukaryotic 23S rRNAs annotated in EMBL (this study) confirms all are encoded in chloroplast and mitochondrial genomes. |
|  | IsrR: Iron stress repressed RNA | RF01419 | Horizontal: bacteria to eukaryotes, photosystem I-associated in cyanobacteria ; distribution of eukaryotic IsrR RNAs annotated in EMBL (this study) confirms all are encoded in chloroplast genomes. |

**Table S2. Distribution of CRISPR crRNA annotations in Rfam**

|  |  | **Bacteria** | | **Archaea** | |
| --- | --- | --- | --- | --- | --- |
| **CLAN** | **RFAM ID** | **Total species** | **Total RNAs** | **Total species** | **Total RNAs** |
| CL00014 | RF01315 | 54 | 5037 | 4 | 123 |
|  | RF01317 | 157 | 3367 | 0 | 0 |
|  | RF01327 | 12 | 1019 | 0 | 0 |
|  | RF01328 | 0 | 0 | 1 | 53 |
|  | RF01338 | 0 | 0 | 7 | 528 |
|  | RF01352 | 6 | 214 | 0 | 0 |
|  | RF01379 | 1 | 128 | 0 | 0 |
| CL00015 | RF01318 | 12 | 608 | 0 | 0 |
|  | RF01320 | 6 | 348 | 6 | 293 |
|  | RF01376 | 1 | 23 | 0 | 0 |
|  | RF01377 | 0 | 0 | 1 | 4 |
| Singleton families | RF01316 | 25 | 731 | 0 | 0 |
|  | RF01319 | 0 | 0 | 7 | 615 |
|  | RF01321 | 4 | 78 | 0 | 0 |
|  | RF01322 | 8 | 197 | 0 | 0 |
|  | RF01323 | 6 | 452 | 0 | 0 |
|  | RF01324 | 0 | 0 | 1 | 38 |
|  | RF01325 | 6 | 213 | 0 | 0 |
|  | RF01326 | 0 | 0 | 2 | 139 |
|  | RF01329 | 2 | 15 | 0 | 0 |
|  | RF01330 | 2 | 51 | 0 | 0 |
|  | RF01331 | 3 | 114 | 0 | 0 |
|  | RF01332 | 5 | 1288 | 0 | 0 |
|  | RF01333 | 2 | 73 | 0 | 0 |
|  | RF01334 | 2 | 548 | 0 | 0 |
|  | RF01335 | 53 | 518 | 0 | 0 |
|  | RF01336 | 2 | 177 | 0 | 0 |
|  | RF01337 | 0 | 0 | 8 | 289 |
|  | RF01339 | 0 | 0 | 1 | 125 |
|  | RF01340 | 1 | 24 | 0 | 0 |
|  | RF01341 | 1 | 19 | 0 | 0 |
|  | RF01342 | 1 | 30 | 0 | 0 |
|  | RF01343 | 5 | 62 | 0 | 0 |
|  | RF01344 | 25 | 74 | 0 | 0 |
|  | RF01345 | 2 | 32 | 0 | 0 |
|  | RF01346 | 4 | 158 | 0 | 0 |
|  | RF01347 | 3 | 34 | 0 | 0 |
|  | RF01348 | 5 | 120 | 0 | 0 |
|  | RF01349 | 3 | 82 | 0 | 0 |
|  | RF01350 | 0 | 0 | 2 | 118 |
|  | RF01351 | 0 | 0 | 2 | 9 |
|  | RF01353 | 1 | 1 | 2 | 69 |
|  | RF01354 | 0 | 0 | 4 | 327 |
|  | RF01355 | 0 | 0 | 4 | 203 |
|  | RF01356 | 6 | 498 | 0 | 0 |
|  | RF01357 | 2 | 37 | 0 | 0 |
|  | RF01358 | 0 | 0 | 2 | 89 |
|  | RF01359 | 1 | 6 | 0 | 0 |
|  | RF01360 | 0 | 0 | 1 | 43 |
|  | RF01361 | 2 | 11 | 0 | 0 |
|  | RF01362 | 9 | 182 | 0 | 0 |
|  | RF01363 | 2 | 48 | 0 | 0 |
|  | RF01364 | 1 | 23 | 0 | 0 |
|  | RF01365 | 1 | 50 | 0 | 0 |
|  | RF01366 | 1 | 14 | 0 | 0 |
|  | RF01367 | 1 | 15 | 0 | 0 |
|  | RF01368 | 1 | 34 | 0 | 0 |
|  | RF01369 | 0 | 0 | 2 | 92 |
|  | RF01370 | 5 | 103 | 0 | 0 |
|  | RF01371 | 2 | 139 | 0 | 0 |
|  | RF01372 | 4 | 18 | 0 | 0 |
|  | RF01373 | 0 | 0 | 3 | 171 |
|  | RF01374 | 16 | 83 | 0 | 0 |
|  | RF01375 | 0 | 0 | 1 | 29 |
|  | RF01378 | 0 | 0 | 2 | 101 |

**Table S3.** Distribution of spliceosomal RNAs across eukaryotes*

|  | Amoebozoa | | Opisthonkonts | | Archaeplastida | | Chromalveolata | | Excavates | | | Rhizaria | |
| --- | --- | --- | --- | --- | --- | --- | --- | --- | --- | --- | --- | --- | --- |
|  | Rfam | DL | Rfam | DL | Rfam | DL | Rfam | DL | Rfam | DL | Chen | Rfam | DL |
| U1 | 7 | x | 10663 | x | 415 | x | 85 | x | 2 | x | x | 0 |  |
| U2 | 22 | x | 6682 | x | 540 | x | 183 | x | 30 | x | x | 0 |  |
| U4 | 4 | x | 5369 | x | 143 | x | 61 | x | 0 | x | x | 0 |  |
| U5 | 9 | x | 2996 | x | 330 | x | 87 | x | 1 | x | x | 0 |  |
| U6 | 9 | x | 47847 | x | 356 | x | 205 | x | 19 | x | x | 0 |  |
| U11 | 0 | x | 393 | x | 29 | x | 4 | x | 0 |  |  | 0 |  |
| U12 | 1 | x | 295 | x | 22 | x | 4 | x | 0 |  |  | 0 |  |
| U4atac | 0 |  | 419 | x | 0 | x | 0 |  | 0 |  |  | 0 |  |
| U6atac | 0 |  | 1560 | x | 60 | x | 4 | x | 0 |  |  | 0 |  |

*Data are derived from this study (Rfam), Davila-Lopez et al. (DL) and Chen et al. (Chen). Crosses (x) denote presence in one or more eukaryote species within the supergroup; Rfam counts are total number of annotations in EMBL, release 100.

**Table S4.** Records associated with RNA families in the Rfam database^a^

| **Rfam** | **EMBL** | **PUB DATE** | **DESCRIPTION** | **Conservation**^b^ |
| --- | --- | --- | --- | --- |
| RF00028 | 2004 | 1990 | Group I catalytic intron | E-B |
| RF01118 | 2008 | 1987 | Pseudoknot of the domain G(G12) of 23S ribosomal RNA | E-B |
| RF01419 | 1989 | 2006 | Antisense RNA which regulates isiA expression | E-B |
| RF01317 | 2006 | N/A | CRISPR RNA direct repeat element | A-B |
| RF01338 | 2007 | N/A | CRISPR RNA direct repeat element | A-B |
| RF00001 | 1992 | 2000 | 5S ribosomal RNA | LUCA |
| RF00002 | 1993 | 1997 | 5.8S ribosomal RNA | LUCA |
| RF00005 | 1994 | 1993 | tRNA | LUCA |
| RF00009 | 1996 | 1998 | Nuclear RNase P | LUCA |
| RF00010 | 1986 | 1998 | Bacterial RNase P class A | LUCA |
| RF00011 | 1996 | 1998 | Bacterial RNase P class B | LUCA |
| RF00017 | 2005 | 2000 | Eukaryotic type signal recognition particle RNA | LUCA |
| RF00023 | 2006 | 1996 | transfer-messenger RNA | LUCA |
| RF00029 | 2002 | 2001 | Group II catalytic intron | LUCA |
| RF00030 | 2005 | 1993 | RNase MRP | LUCA |
| RF00059 | 2007 | 2001 | TPP riboswitch (THI element) | LUCA |
| RF00169 | 1995 | 2002 | Bacterial signal recognition particle RNA | LUCA |
| RF00177 | 1991 | N/A | Small subunit ribosomal RNA, 5' domain | LUCA |
| RF00373 | 1991 | 1998 | Archaeal RNase P | LUCA |

^a^Displayed are the oldest dates from the literature references (PUB DATE) contained in the corresponding Stockholm file and from the EMBL accessions. Some of these RNAs were discovered experimentally prior to the dates associated with the deposited sequences, meaning the age of many of the oldest RNAs is in fact underestimated. Consequently, the discovery dates summarized in Figure S3 are estimates that can only be used in the context of broad discovery trends.

^b^Abbreviations: present in eukaryotes & bacteria (E-B); present in archaea & bacteria (A-B); present in archaea, bacteria & eukaryotic domains (LUCA).

**References**

1. Illergard K, Ardell DH, Elofsson A (2009) Structure is three to ten times more conserved than sequence--a study of structural response in protein cores. Proteins 77: 499-508.

2. Freyhult EK, Bollback JP, Gardner PP (2007) Exploring genomic dark matter: a critical assessment of the performance of homology search methods on noncoding RNA. Genome Res 17: 117-125.

3. Gardner PP, Daub J, Tate J, Moore BL, Osuch IH, et al. (2011) Rfam: Wikipedia, clans and the "decimal" release. Nucleic Acids Res 39: D141-145.

4. Durbin R, Eddy SR, Krog A, Mitchison G (1998) Biological Sequence Analysis: Probabilistic Models of Proteins and Nucleic Acids: Cambridge University Press.

5. Hoeppner MP, Poole AM (2012) Comparative genomics of eukaryotic small nucleolar RNAs reveals deep evolutionary ancestry amidst ongoing intragenomic mobility. BMC Evol Biol 12: 183.

6. Tatusov RL, Natale DA, Garkavtsev IV, Tatusova TA, Shankavaram UT, et al. (2001) The COG database: new developments in phylogenetic classification of proteins from complete genomes. Nucleic Acids Res 29: 22-28.

7. Finn RD, Mistry J, Tate J, Coggill P, Heger A, et al. (2010) The Pfam protein families database. Nucleic Acids Res 38: D211-222.

8. Sintchak MD, Arjara G, Kellogg BA, Stubbe J, Drennan CL (2002) The crystal structure of class II ribonucleotide reductase reveals how an allosterically regulated monomer mimics a dimer. Nature structural biology 9: 293-300.

9. Madera M (2008) Profile Comparer: a program for scoring and aligning profile hidden Markov models. Bioinformatics 24: 2630-2631.

10. Cuff AL, Sillitoe I, Lewis T, Clegg AB, Rentzsch R, et al. (2011) Extending CATH: increasing coverage of the protein structure universe and linking structure with function. Nucleic Acids Res 39: D420-426.

11. Andreeva A, Howorth D, Chandonia JM, Brenner SE, Hubbard TJ, et al. (2008) Data growth and its impact on the SCOP database: new developments. Nucleic Acids Res 36: D419-425.

12. Fitch WM (2000) Homology a personal view on some of the problems. Trends Genet 16: 227-231.

13. Altenhoff AM, Dessimoz C (2009) Phylogenetic and functional assessment of orthologs inference projects and methods. PLoS Comput Biol 5: e1000262.

14. Weinberg Z, Wang JX, Bogue J, Yang J, Corbino K, et al. (2010) Comparative genomics reveals 104 candidate structured RNAs from bacteria, archaea, and their metagenomes. Genome Biol 11: R31.

15. Haas BJ, Zody MC (2010) Advancing RNA-Seq analysis. Nat Biotechnol 28: 421-423.

16. Rest JS, Mindell DP (2003) Retroids in archaea: phylogeny and lateral origins. Mol Biol Evol 20: 1134-1142.

17. Dai L, Zimmerly S (2003) ORF-less and reverse-transcriptase-encoding group II introns in archaebacteria, with a pattern of homing into related group II intron ORFs. RNA 9: 14-19.

18. Lambowitz AM, Zimmerly S (2004) Mobile group II introns. Annu Rev Genet 38: 1-35.

19. Sun FJ, Caetano-Anolles G (2008) Evolutionary patterns in the sequence and structure of transfer RNA: early origins of archaea and viruses. PLoS Comput Biol 4: e1000018.

20. Maizels N, Weiner AM (1999) The genomic tag hypothesis: what molecular fossils tell us about the evolution of tRNA. In: Gesteland RF, Cech TR, Atkins JF, editors. The RNA world, 2nd ed. 2nd ed ed. Cold Spring Harbor, NY: Cold Spring Harbor Laboratory Press. pp. 79–111.

21. Di Giulio M (1999) The non-monophyletic origin of the tRNA molecule. J Theor Biol 197: 403-414.

22. Rodin AS, Szathmary E, Rodin SN (2011) On origin of genetic code and tRNA before translation. Biology Direct: in press.

23. Enright AJ, Van Dongen S, Ouzounis CA (2002) An efficient algorithm for large-scale detection of protein families. Nucleic Acids Res 30: 1575-1584.

24. Wilkinson M, McInerney JO, Hirt RP, Foster PG, Embley TM (2007) Of clades and clans: terms for phylogenetic relationships in unrooted trees. Trends Ecol Evol 22: 114-115.

25. Haugen P, Simon DM, Bhattacharya D (2005) The natural history of group I introns. Trends Genet 21: 111-119.

26. Cedergren R, Gray MW, Abel Y, Sankoff D (1988) The evolutionary relationships among known life forms. J Mol Evol 28: 98-112.

27. Duhring U, Axmann IM, Hess WR, Wilde A (2006) An internal antisense RNA regulates expression of the photosynthesis gene isiA. Proc Natl Acad Sci USA 103: 7054-7058.

28. Woese CR, Kandler O, Wheelis ML (1990) Towards a natural system of organisms: proposal for the domains Archaea, Bacteria, and Eucarya. Proc Natl Acad Sci USA 87: 4576-4579.

29. Sun FJ, Caetano-Anolles G (2009) The evolutionary history of the structure of 5S ribosomal RNA. J Mol Evol 69: 430-443.

30. Sun FJ, Caetano-Anolles G (2010) The ancient history of the structure of ribonuclease P and the early origins of Archaea. BMC Bioinformatics 11: 153.

31. Collins LJ, Moulton V, Penny D (2000) Use of RNA secondary structure for studying the evolution of RNase P and RNase MRP. Journal of molecular evolution 51: 194-204.

32. Schmitz U, Behrens S, Freymann DM, Keenan RJ, Lukavsky P, et al. (1999) Structure of the phylogenetically most conserved domain of SRP RNA. RNA 5: 1419-1429.

33. Zwieb C, van Nues RW, Rosenblad MA, Brown JD, Samuelsson T (2005) A nomenclature for all signal recognition particle RNAs. RNA (New York, NY 11: 7-13.

34. Kunin V, Sorek R, Hugenholtz P (2007) Evolutionary conservation of sequence and secondary structures in CRISPR repeats. Genome Biol 8: R61.

35. Shah SA, Garrett RA (2011) CRISPR/Cas and Cmr modules, mobility and evolution of adaptive immune systems. Res Micro 162: 27-38.

36. Nikoh N, Fukatsu T (2001) Evolutionary dynamics of multiple group I introns in nuclear ribosomal RNA genes of endoparasitic fungi of the genus Cordyceps. Mol Biol Evol 18: 1631-1642.

37. Cannone JJ, Subramanian S, Schnare MN, Collett JR, D'Souza LM, et al. (2002) The comparative RNA web (CRW) site: an online database of comparative sequence and structure information for ribosomal, intron, and other RNAs. BMC Bioinformatics 3: 2.

38. Perotto S, Nepote-Fus P, Saletta L, Bandi C, Young JP (2000) A diverse population of introns in the nuclear ribosomal genes of ericoid mycorrhizal fungi includes elements with sequence similarity to endonuclease-coding genes. Mol Biol Evol 17: 44-59.

39. Davila Lopez M, Rosenblad MA, Samuelsson T (2008) Computational screen for spliceosomal RNA genes aids in defining the phylogenetic distribution of major and minor spliceosomal components. Nucleic Acids Res 36: 3001-3010.

40. Chen XS, White WT, Collins LJ, Penny D (2008) Computational identification of four spliceosomal snRNAs from the deep-branching eukaryote Giardia intestinalis. PLoS One 3: e3106.
